# Elevated levels of cerebrospinal fluid α-synuclein oligomers in healthy asymptomatic *LRRK2* mutation carriers

**DOI:** 10.3389/fnagi.2014.00248

**Published:** 2014-09-25

**Authors:** Jan O. Aasly, Krisztina K. Johansen, Gunnar Brønstad, Bjørg J. Warø, Nour K. Majbour, Shiji Varghese, Fatimah Alzahmi, Katerina E. Paleologou, Dena A. M. Amer, Abdulmonem Al-Hayani, Omar M. A. El-Agnaf

**Affiliations:** ^1^Department of Neuroscience, Norwegian University of Science and Technology (NTNU)Trondheim, Norway; ^2^Department of Neurology, St. Olav’s Hospital, University Hospital of TrondheimTrondheim, Norway; ^3^Department of Biochemistry, College of Medicine and Health Sciences, United Arab Emirates UniversityAl Ain, United Arab Emirates; ^4^Department of Molecular Biology and Genetics, Democritus University of ThraceAlexandroupolis, Greece; ^5^Department of Anatomy, Faculty of Medicine, King Abdulaziz UniversityJeddah, Saudi Arabia; ^6^Faculty of Medicine, King Abdulaziz UniversityJeddah, Saudi Arabia

**Keywords:** Parkinson’s disease, LRRK2 mutation carriers, CSF, biomarkers, alpha-synuclien

## Abstract

Mutations in the leucine-rich repeat kinase 2 gene are the most common cause of autosomal dominant Parkinson’s disease (PD). To assess the cerebrospinal fluid (CSF) levels of α-synuclein oligomers in symptomatic and asymptomatic leucine-rich repeat kinase 2 mutation carriers, we used enzyme-linked immunosorbent assays (ELISA) to investigate total and oligomeric forms of α-synuclein in CSF samples. The CSF samples were collected from 33 Norwegian individuals with leucine-rich repeat kinase 2 mutations: 13 patients were clinically diagnosed with PD and 20 patients were healthy, asymptomatic leucine-rich repeat kinase 2 mutation carriers. We also included 35 patients with sporadic PD (sPD) and 42 age-matched healthy controls. Levels of CSF α-synuclein oligomers were significantly elevated in healthy asymptomatic individuals carrying leucine-rich repeat kinase 2 mutations (*n* = 20; *P* < 0.0079) and in sPD group (*n* = 35; *P* < 0.003) relative to healthy controls. Increased α-synuclein oligomers in asymptomatic leucine-rich repeat kinase 2 mutation carriers showed a sensitivity of 63.0% and a specificity of 74.0%, with an area under the curve of 0.66, and a sensitivity of 65.0% and a specificity of 83.0%, with an area under the curve of 0.74 for sPD cases. An inverse correlation between CSF levels of α- synuclein oligomers and disease severity and duration was observed. Our study suggests that quantification of α-synuclein oligomers in CSF has potential value as a tool for PD diagnosis and presymptomatic screening of high-risk individuals.

## Introduction

Parkinson’s disease (PD) is the most common age-related movement disorder and the second most common neurodegenerative disorder after Alzheimer’s disease. The earliest clinical features of PD are typically retrospective and not specific, and they may include depression, hyposmia, constipation, and sleep disorders. At least 70% of the neurons in the substantia nigra (SN) are lost prior to the appearance of any major motor symptoms (Schapira, 1999). The main motor symptoms, including resting tremor, rigidity, bradykinesia, and postural instability, are collectively known as parkinsonism. Currently, PD is clinically diagnosed and clinical trials of disease-modifying drugs are initiated only after most of the vulnerable dopaminergic neurons in the SN have already been lost. Individuals at risk for PD with less complete loss of dopaminergic neurons may be more responsive to and benefit most from neuroprotective therapies. Therefore, identifying biomarkers for early diagnosis may facilitate the development of novel treatments designed to slow disease progression. Furthermore, such findings may help to elucidate the pathophysiology of PD. Most PD cases are sporadic (sPD) (i.e., idiopathic; attributed to unknown causes). However, some atypical cases involve genetic susceptibility (Singleton et al., 2013). Over the last two decades, several genetic causes of PD have been identified. At present, 5–10% of all PD cases can be traced to a known genetic cause that is either monogenic or related to a combination of susceptibility factors (Singleton et al., 2013). Mutations in the gene encoding α-synuclein (α-syn) (*SNCA*) were the first genetic factors to be linked to familial PD (Polymeropoulos et al., 1997; Krüger et al., 1998; Singleton et al., 2003). Although *SNCA* mutations rarely cause late-onset familial PD, *SNCA* is still of great importance to PD etiology, as abnormal aggregation of α-syn in the brain is also found in neuropathological lesions (Lewy bodies (LBs); Spillantini et al., 1997). However, it has been previously shown that α-syn is normally released by neuronal cells and present in the cerebrospinal fluid (CSF) and peripheral plasma (El-Agnaf et al., 2003) Recent studies have demonstrated that oligomeric forms of α-syn are neurotoxic species *in vitro* and *in vivo*, whereas amyloid fibrils may not be directly toxic (Winner et al., 2011). Gene mutations in leucine-rich repeat kinase 2 (LRRK2) are the second most common cause of autosomal dominant PD and cause 2–5% of familial PD. The most common point mutation, G2019S, has been shown to be involved in 5–6% of autosomal dominant PD cases (Di Fonzo et al., 2005; Nichols et al., 2005; Dächsel and Farrer, 2010) and 1–2% of sPD cases (Gilks et al., 2005). Patients with late-onset monogenic forms of PD may demonstrate subtle signs or symptoms several years before they suffer from any motor symptoms (Sossi et al., 2010; Johansen et al., 2011; Ruiz-Martínez et al., 2011). Similarly, recent positron emission tomography (PET) studies have confirmed dopaminergic dysfunction in asymptomatic LRRK2 mutation carriers (Nandhagopal et al., 2008). Therefore, these carriers are an ideal population for identifying novel biomarkers for the early diagnosis of PD. We and other groups recently reported elevated levels of α-syn oligomers (o-α-syn) and an increased o-α-syn/total-α-syn (t-α-syn) ratio in CSF from PD patients relative to controls (Tokuda et al., 2010; Park et al., 2011; Sierks et al., 2011; Parnetti et al., 2014a,b). These findings suggest that CSF α-syn oligomers could be a potentially useful biomarker for diagnosis and possible early detection of PD. We therefore explored the potential use of o-α-syn as an early biomarker for PD in CSF from asymptomatic LRRK2 mutation carriers and symptomatic LRRK2 PD patients relative to sPD patients and healthy age-matched controls.

## Materials and methods

### Patient population and clinical methods

In total, 33 Norwegian individuals from 12 different families with mutant LRRK2 were assessed in this study. Thirteen patients were clinically diagnosed with PD and 20 patients were healthy, asymptomatic LRRK2 mutation carriers. These families have been extensively described in previous report (Aasly et al., 2005, 2010; Johansen et al., 2010). In addition, 35 patients with sPD and 42 age-matched healthy controls were also recruited for this study from St. Olav’s Hospital at the University Hospital of Trondheim in Norway. Parkinson’s disease was diagnosed according to established diagnostic criteria (Gelb vs. UK Parkinson’s Disease Society). Disease severity was defined according to the Hoehn and Yahr scale (H&Y). All patients with sPD were screened and tested negative for known *LRRK2* mutations. Patients with age at onset ≤50 years also tested negative for known pathogenic mutations in *Parkin* and *PINK1*. All family members were screened for clinical signs of PD and found to be asymptomatic, although a few had mild pre-motor signs with an increased Unified Parkinson’s Disease Rating Scale (UPDRS) score (Johansen et al., 2011). The LRRK2-mutant PD patients were on levodopa, and some were taking other dopamine agonists and monoamine oxidase-B (MAO-B) inhibitors. The mean levodopa-equivalent dose in the LRRK2-mutant PD group varied between 300 and 1800 mg, with a mean of 580 ± 422 mg, and the mean levodopa-equivalent dose in the sPD group was 300 to 1500 mg, with a mean of 628 ± 387 mg.

All individuals underwent lumbar puncture between 08:00 am and 10:00 am following overnight fasting. A small sample of CSF was sent for routine analysis, and then 18 to 22 ml was sampled and frozen in 15 aliquots of 1.2–1.5 ml each within 15 min of completion of the lumbar puncture. The aliquots were stored at −80°C until further analysis. All patients gave signed, informed consent, and the study was approved by the Regional Committee for Medical and Health Research Ethics.

### Size Exclusion Chromatography (SEC) for separating α-syn monomers and oligomers

Size Exclusion Chromatography was carried out using an AKTA FPLC system (GE Healthcare-Sweden) and a superdex 200 column at 4°C. Concentrated 0.5 ml of CSF were loaded onto the column and eluted with PBS (pH 7.4) at a flow rate of 0.1 ml/min (0.5 ml/fraction). The elution of α-syn was monitored at absorbance wavelengths of 215 nm. Fractions of 1 ml were collected, concentrated to 100 μl using a speed vac, and analyzed by the western blotting for the presence of α-syn. To determine the elution time of monomeric and oligomeric α-syn, molecular weight standards (Thyroglobulin 669 kDa, ferritin 440 kDa, aldolase 171 kDa, abmumin 68 kDa and chymotrypsinogenA 25 kDa), fresh α-syn solution and aged α-syn solution were co-injected into the column and eluted at the same conditions mentioned above.

### Sodium dodecyl sulfate–polyacrylamide gel electrophoresis (SDS–NuPAGE) and immunoblotting

The CSF fractions were separated on NuPAGE Bis–Tris 4–12%, 1 mm gels (Invitrogen Ltd., Paisley, UK) and then transferred to nitrocellulose membranes (0.45 μm) at 30 V, 125 mA for 45 min (Invitrogen Ltd., Paisley, UK). Membranes were boiled for 5 min in PBS then blocked for 1 h with 5% marvel dried skimmed milk and dissolved in PBS–Tween 20 (0.05%) (PBST). The membranes were probed overnight at 4°C anti-α-syn (211) mouse monoclonal antibody to α-syn (aa 121–125). The membranes were washed several times with PBST followed by incubation with horseradish peroxidase (HRP)-conjugated goat anti-mouse (Dako Ltd., Ely, UK), for 60 min. The membranes were again washed several times with PBST. The protein bands were visualized using ECL reagents (Pierce, USA) as described by the manufacturer.

### Immunoassays for total and oligomeric α-synuclein in CSF

A sandwich enzyme-linked immunosorbent assay (ELISA) methods were employed to measure total or oligomeric α-syn levels in CSF samples as described previously (Tokuda et al., 2010).

### Statistical analysis

Differences between groups were compared using a Mann-Whitney U test. Significance was defined as *P* < 0.05. Correlational analysis was conducted by Pearson simple correlation. The receiver operating characteristic (ROC) was analyzed to assess the most appropriate cut-off values for the level of CSF α-syn oligomer and the oligomers/total-α-syn ratio in the CSF to distinguish between groups. All analyses were conducted using GraphPad Prism software (GraphPad Prism Version 4.0, GraphPad software, San Diego, CA).

## Results

### Patient population and demographics

In total, 33 Norwegian individuals from 12 different *LRRK2* families were investigated in the present study. Thirteen individuals with *LRRK2* point mutations had developed symptomatic PD, including 11 males who were carrying the most common *LRRK2* point mutation, G2019S, and two females who were carrying a different *LRRK2* point mutation, N1437H. The 13 individuals had a mean age of 64.0 years ± 13.3 years. In contrast, 20 individuals were healthy asymptomatic *LRRK2* mutation carriers [G2019S (*n* = 16) and N1437H (*n* = 4)]. These 20 individuals had a mean age of 55.4 years ± 15 years. None of the healthy asymptomatic *LRRK2* mutation carriers (LRRK2-H) had any complaints of a movement disorder. Some were receiving medication for diabetes mellitus, mild hypertension, and other minor health problems. In addition, 35 patients with sPD and 42 age-matched healthy controls were also included in this study. No significant difference was noticed in disease duration between symptomatic PD patients with *LRRK2* mutations (LRRK2-PD) and sPD patients. Moreover, there was no difference between the groups with regard to CSF levels of leukocytes or total protein, albumin, and glucose levels, including plasma glucose levels. Controling for age and gender did not significantly alter the results in any case. A summary of the patient population employed in the present study and the respective demographic details are shown in Table 1.

**Table 1:** **Details of patient population employed in the present study and the demographics**.

Groups	Symptomatic PD due to LRRK2 mutations	Asymptomatic LRRK2 mutation carriers	Sporadic PD	Healthy controls
**Number of individuals**	13	20	35	42
**Gender (M/F)**	11/2	11/9	23/2	14/21
**Age range (y)**	43–87	26–76	38–71	37–74
**Mean Age (y)**	64 ± 13.3	55.4 ± 15	54 ± 15	59 ± 10
**Levodopa equivalents**	580 ± 422 mg	NA	628 ± 387 mg	NA
**H-Y grade**	2.7 ± 0.7	NA	2.28 ± 0.6	NA
**Disease duration (months)**	24–330	NA	12–300	NA

*PD = Parkinson’s disease; y = years; M = male; F = female; H-Y grade = Hoehn-Yahr grade*.

### Levels of total α syn (t-α syn) in CSF samples

To measure the total α-syn (t-α-syn) in CSF samples, we recently optimized our original α-syn ELISA protocol using a chemiluminescence-based read-out arm for HRP-labeled antibody detection (Tokuda et al., 2010). We demonstrated that our optimized protocol yielded excellent performance with regard to both specificity and sensitivity. Using this system, an increase of approximately 100-fold in the detection of recombinant α-syn was recorded, ranging from 0.010 to >500 ng/ml (Tokuda et al., 2010).

**Figure 1:**
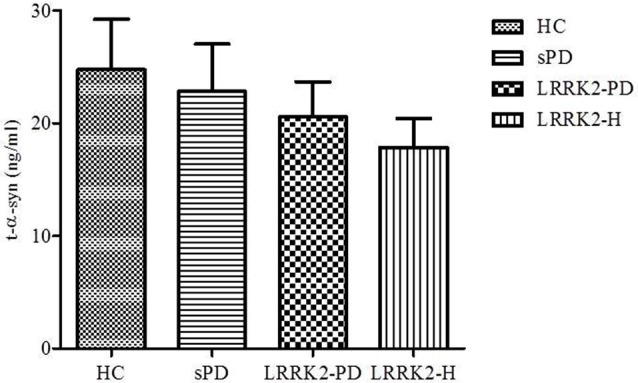
**Levels of t-α-syn (in ng/ml) in CSF from healthy controls (HC), sporadic PD patients (sPD), symptomatic PD patients with LRRK2 mutations (LRRK2-PD), and healthy asymptomatic LRRK2 mutation carriers (LRRK2-H)**. Values are expressed as the mean ± standard deviation.

As illustrated in Figure 1, the concentration of t-α-syn varied considerably among the four studied groups, although the difference was not statistically significant. Lower mean concentrations of CSF t-α-syn were observed in patients with sPD (mean ± SEM = 22.81 ± 4.198 ng/ml, *n* = 35), LRRK2-PD (mean ± SEM = 20.54 ± 3.139 ng/ml, *n* = 13), and LRRK2-H (mean ± SEM = 17.84 ± 2.569 ng/ml, *n* = 20) than in age-matched controls (mean ± SEM = 24.74 ± 4.470 ng/ml, *n* = 42) (*P* = 0.7001, Mann-Whitney U-test).

### CSF α syn oligomers (o-α syn) levels

**Figure 2:**
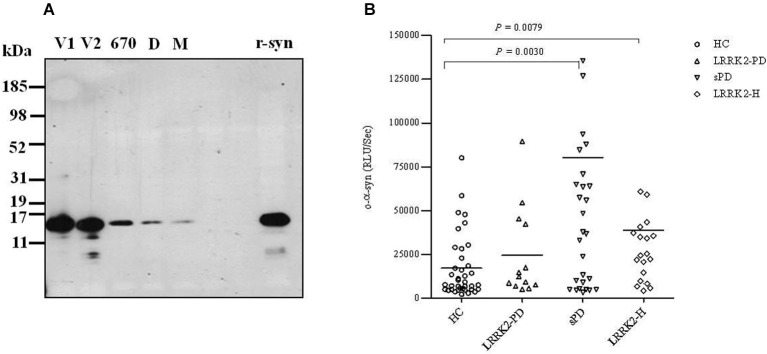
**(A)** Size exclusion chromatographic analysis of α-syn oligomers in CSF. Five mls of CSF from a PD patient concentrated to 0.5 ml, was fractionated on a superdex 200 SE column. The elution volume for monomeric (M) α-syn was determined by fresh recombinant α-syn solution and molecular weight standard, and was eluted in a peak corresponding to column volume of 13–14 ml (kDa ~68), while dimeric (D) α-syn was eluted in a peak corresponding to column volume of approximately 12 ml (kDa ~170). High molecular weight oligomeric α-syn was determined by aged recombinant α-syn solution and molecular weight standard, and was eluted in a peak corresponding to column volume of approximately 9 ml (670 kDa). The void volume (V) corresponds to MW >670 kDa. The fractions of 1 ml which correspond to the oligomeric and monomeric α-syn peaks were separately collected, concentrated to 100 μl using a speed vac, and then analyzed by western blotting for the presence of α-syn using anti-α-syn (211) antibody (1:1000). **(B)** Scatter plot representing individual values of the levels of o-α-syn (in relative luminescence units/second [RLU/s]) in CSF from healthy controls (HC; open circles), symptomatic PD patients with LRRK2 point mutations (LRRK2-PD; open triangles), patients with (sPD; inverted open triangles), and healthy asymptomatic LRRK2 mutation carriers (LRRK2-H; open squares). Each bar represents the mean value.

After measuring the total α-syn in CSF samples from our cohort, we then measured the levels of CSF α-syn oligomers (o-α-syn) in the same samples. For such measurements, we used an ELISA system for α-syn oligomers that specifically detects soluble oligomers without detecting monomeric forms of α-syn (El-Agnaf et al., 2006). Because α-syn oligomers represent a small fraction of the total α-syn in CSF, for this assay we also used a chemiluminescence-based read-out arm to allow detection of low levels of CSF α-syn oligomers. The ELISA protocol was based on a conventional sandwich system in which mAb 211 was used to capture α-syn, followed by detection with a biotinylated form of 211. Subsequently, the biotinylated mAb was detected with ExtrAvidin-HRP, followed by a chemiluminescent substrate. In this assay, no signal is detected for monomeric α-syn, as the capture mAb occupies the only available antibody binding site on the protein. In contrast, multiple mAb binding sites are available in the case of oligomeric forms of α-synuclein, thus permitting both capture and detection (El-Agnaf et al., 2006). This assay has been extensively characterized and yields excellent performance in both specificity and sensitivity (Tokuda et al., 2010). In order to investigate the size of α-syn oligomers detected by our ELISA in CSF, recently we used SEC to fractionate fresh CSF samples from those PD patients that gave a robust signal in our ELISA. The western blot revealed immunoreactive material with an elution peak in SEC fractions corresponding to MW of 70 and 170 kDa which belong to α-syn monomers and dimmers respectively. However, much of the immunereactive material was eluted at the void volume, which indicated a MW of >670 kDa (Figure 2A). These SEC fractionation results support the notion that the CSF from PD patients mainly contain HMW α-syn oligomers, since most of the protein material detected by the ELISA has a MW >760 kDa. Experiments are in progress in our laboratory to further characterize and analyze the structure and nature of the oligomeric species of α-syn and to discern any modifications of α-syn detected by the ELISA (i.e., nitrated, phosphorylated, etc.). This information will be useful in order to improve both the sensitivity and selectivity for α-syn protein species in our future ELISA variants.

A scatter plot representing the CSF levels of o-α-syn for each patient in the four groups is shown in Figure 2B; the level of o-α-syn is given as the chemiluminescence signal intensity (relative luminescence units/second [RLU/s]). The level of CSF o-α-syn was significantly higher by 4-fold in the sPD group (mean ± SEM = 80,186 ± 23,861, *n* = 35) relative to the healthy age-matched control group (mean ± SEM = 17,117 ± 2943, *n* = 42) (*P* < 0.01, Mann-Whitney U test). Significantly higher levels of CSF o-α-syn (approximately 2-fold higher) were also detected in LRRK2-H (mean ± SEM = 38,754 ± 12,514, *n* = 20) relative to the control group (mean ± SEM = 17,117 ± 2,943, *n* = 42) (*P* < 0.01, Mann-Whitney U test). Unexpectedly, LRRK2-PD patients (mean ± SEM = 24,510 ± 7,161, *n* = 13) did not show any significant difference in the level of o-α-syn in the CSF relative to the healthy control group (*P* = 0.1910, Mann-Whitney U test), possibly because of the low number of individuals in this group (*n* = 13 vs. *n* = 42). Furthermore, we observed a significant inverse correlation (Spearman *r* = −0.49, *P* < 0.05) between the severity of the disease and the level of o-α-syn in the CSF of sPD patients using H&Y grading (Table 2). However, no significant correlation (Spearman *r* = −0.5391, *P* = 0.09) between H&Y grading and the levels of o-α-syn in the CSF of LRRK2-PD patients was observed (Table 2). Interestingly, when sPD and LRRK2-PD cases were combined together, more significant and stronger inverse correlation was noted (Spearman *r* = −0.6, *P* = 0.0004, Table 2). Whereas, CSF t-α-syn levels did not correlate with H&Y levels. In parallel, statistically significant inverse correlation between CSF o-α-syn and disease duration was also observed in sPD group (Spearman *r* = −0.45, *P* < 0.05), but CSF o-α-syn did not correlate with disease duration in LRRK2-PD group. Whereas, we observed significant negative correlation between CSF o-α-syn levels and disease duration when the sPD and LRRK2-PD groups were combined together (Spearman *r* = −0.5, *P* = 0.002, testable 2). In contrast, CSF o-α-syn levels did not correlate with UPDRS scores within any of the groups (data not shown). In addition, no correlation was observed between the level of o-α-syn in the CSF and the age of the patients (data not shown).

**Table 2:** **Spearman correlations between CSF α-syn species, disease duration (months), and Hoehn and Yahr stage in sPD and LRRK2-PD**.

	Disease duration (months)			Hoehn and Yahr stage		
Case	sPD	LRRK2-PD	sPD+LRRK2-PD	sPD	LRRK2-PD	sPD+LRRK2-PD
t-α-syn	NS	NS	NS	NS	NS	NS
o-α-syn	−0.45*	NS	−0.5**	−0.5*	NS	−0.6***

* *P* < 0.05, ** *P* < 0.01, *** *P* < 0.001, NS = Not-Significant.

**Figure 3:**
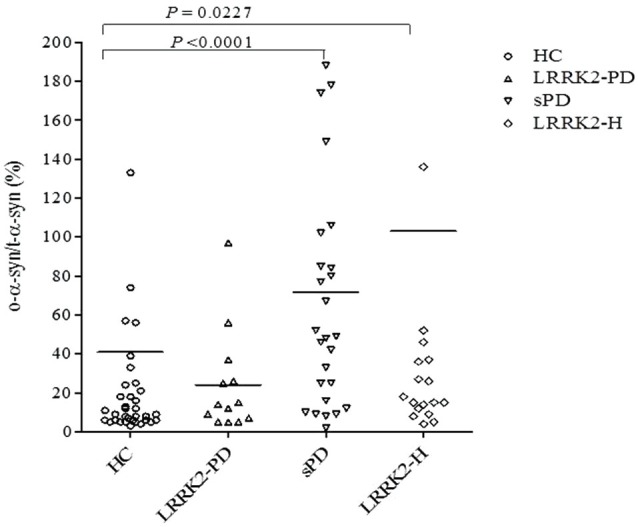
**Scatter plot presenting individual values for the ratio of o-α-syn to t-α-syn (o-α-syn/t-α-syn ratio,%) in CSF from healthy controls (HC; open circles), symptomatic PD patients with LRRK2 point mutations (LRRK2-PD; open triangles), patients with (sPD; inverted open triangles), and healthy asymptomatic LRRK2 mutation carriers (LRRK2-H; open squares)**. Each bar represents the mean value. The *P* value for each regression line is shown in each subfigure.

The measurement of both total and oligomeric α-syn in RLU/s allowed us to calculate the ratio of o-α-syn to t-α-syn (o-α-syn/t-α-syn ratio [%]) in the CSF of each patient, as shown in Figure 3. This ratio was found to be significantly higher in the sPD group (mean ± SEM = 71.59 ± 12.76, *n* = 35) (*P* < 0.001, Mann-Whitney U test) and LRRK2-H group (mean ± SEM = 103.1 ± 47.91, *n* = 20) (*P* < 0.05, Mann-Whitney U test) than in the healthy control group (mean ± SEM = 40.97 ± 21.62, *n* = 42). No significant difference in the ratio of o-α-syn to t-α-syn was found in the LRRK2-PD relative to controls (mean ± SEM = 24.08 ± 7.359, *n* = 13) (*P* = 0.5297, Mann-Whitney U test), possibly because of the small number of individuals in this group (*n* = 13 vs. *n* = 42).

**Figure 4:**
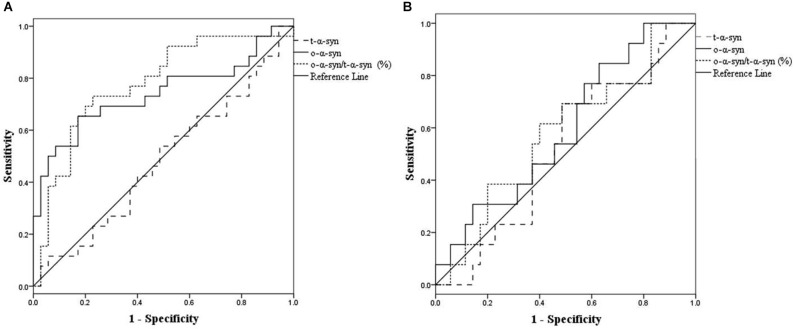
**Use of receiver operating characteristic (ROC) curves for the levels of CSF t-α-syn, o-α-syn and o-α-syn/t-α-syn ratio (%) to discriminate between (sPD) patients and controls (A), and healthy asymptomatic LRRK2 mutation carriers (LRRK2-H) patients and controls (B)**.

Figure 4 shows the ROC curve for CSF o-α-syn and the ratio of o-α-syn to t-α-syn in the discrimination of PD patients from controls. The ROC curve indicates that cutoff values of 31,671.9 RLU/s for CSF o-α-syn and 24.1% for the CSF o-α-syn to t-α-syn ratio were the most reliable measures to distinguish sPD from controls. Detection of CSF α-syn oligomers yielded a sensitivity of 65% (95% CI, 53–77%) and a specificity of 83% (95% CI, 73–93%), with an area under the curve (AUC) of 0.74 (95% CI, 0.60–0.88). The o-α-syn to t-α-syn ratio yielded a sensitivity of 73% (95% CI, 62 to 84%) and a specificity of 77% (95% CI, 0.66–0.88), with an AUC of 0.79 (95% CI, 0.67–0.91). Furthermore, the ROC analysis demonstrated that cutoff values of 20,566 RLU/s for CSF o-α-syn and 9.4% for the CSF o-α-syn to t-α-syn ratio provided the most reliable measure to differentiate LRRK2-H from healthy controls. The sensitivity and specificity of CSF o-α-syn to predict LRRK2-H were 63% (95% CI, 0.50–0.76) and 74% (95% CI, 0.62–0.86), respectively, with an AUC of 0.66 (95% CI, 0.50 to 0.82). The sensitivity of the CSF oligomer to t-α-syn ratio was 53% (95% CI, 39–67%), and the specificity was 81% (95% CI, 0.70–0.92) with an AUC of 0.64 (95% CI, 0.47–0.80).

## Discussion

Currently the diagnosis of PD is based mainly on clinical symptoms. However, differential diagnosis from other parkinsonisms can be difficult and can lead to misdiagnosis. To date, no simple laboratory biomarker is available to detect individuals at risk for PD before most of their dopaminergic neurons have been lost.

Numerous studies have suggested that neuronal cell death may result from the formation of oligomeric species of α-syn in the brain (Irvine et al., 2008). It has been previously shown that levels of soluble o-α-syn are elevated in brain homogenates from patients with PD and DLB relative to normal brains (Paleologou et al., 2009). Recently, it has been reported by us and others significant higher levels of CSF o-α-syn in PD patients compared to age-matched controls (Tokuda et al., 2010; Park et al., 2011; Sierks et al., 2011; Parnetti et al., 2014a,b). Levels of o-α-syn and the o-α-syn to t-α-syn ratio have also been shown to be higher in patients with mild PD (H&Y grades 1 and 2) and patients with early PD (within 24 months after onset) relative to a control group (Tokuda et al., 2010). The aim of this study was to determine whether o-α-syn is suitable biomarker for detecting PD at the early stages of the disease. Recently, abnormal PET changes and olfactory dysfunction were reported in LRRK2-H (Nandhagopal et al., 2008; Ruiz-Martínez et al., 2011; Saunders-Pullman et al., 2011). Therefore, healthy family members with *LRRK2* mutations are an excellent population for validating surrogate biomarkers for early stages of PD. A recent study reported a lack of a statistically significant relationship between PET scan evidence of lost striatal dopaminergic function and the levels of DJ-1 and t-α-syn in CSF from LRRK-H or LRRK-PD cases (Shi et al., 2012). In the present study, we assessed the levels of CSF o-α-syn in symptomatic and asymptomatic *LRRK2* mutation carriers and in sPD cases. We observed significantly elevated levels of CSF α-syn oligomers in LRRK-H relative to healthy controls (Figure 2B), which suggests that the formation of o-α-syn in the brain commences several years before *LRRK2* mutation carriers experience any motor symptoms of PD. Furthermore, we also observed significantly higher o-α-syn levels in the CSF of sPD cases relative to healthy age-matched controls (Figure 2B), which confirms previous findings (Tokuda et al., 2010; Park et al., 2011; Sierks et al., 2011). Unexpectedly, we did not observe any significant difference in the CSF levels of o-α-syn between LRRK-PD patients and healthy controls, possibly because of the low number of individuals in this group (*n* = 13 vs. *n* = 42). However, we noticed in this group that patients with mild PD (H&Y grade ≤2) showed high levels of CSF o-α-syn similar to those of sPD patients. Conversely, patients with higher H&Y grades (>2) had lower levels of α-syn oligomers, which supports the hypothesis that at the early stages of the disease, high levels of toxic o-α-syn accumulate in the brain. In support of this notion, we also found that levels of CSF o-α-syn were inversely correlated with disease duration and H&Y grade in LRRK2-PD and sPD cases, which confirms that CSF levels of o-α-syn decrease with increasing PD severity (Table 2). Thus, we speculate that o-α-syn is formed during the early stages of the disease prior to any major clinical manifestation.

Unified Parkinson’s Disease Rating Scale is the most commonly used clinical scale to provide an efficient and flexible assessment of motor performance in PD patients and to monitor the degree of resultant disability. However, thus far, no strong linear relationship has been established between UPDRS scores and the progressive nigrostriatal degeneration in PD, which may underlie the absence of a correlation between CSF α-syn levels and UPDRS scores. Cerebrospinal fluid biomarkers mirror changes within the brain as an entire unit, whereas UPDRS scores primarily reflect changes in the nigrostriatal dopaminergic system. In addition, compensatory responses in PD may further confound the correlation between CSF biomarkers and the severity of PD motor symptoms (Shi et al., 2011). Moreover, DA replacement therapy provided to PD patients enhances motor function while showing little or no effect on CSF protein concentrations (Hong et al., 2010; Shi et al., 2011).

It has been recently reported that the most common *LRRK2* point mutation, G2019S, initiates and enhances the formation of α-syn aggregates (Lin et al., 2009), possibly by impairing degradation pathways such as the autophagy-lysosomal pathway (Ferree et al., 2012; Tong et al., 2012). Overall, the potential interactions of LRRK2 and α-syn have not been clearly established. Although most *LRRK2*-related PD cases are pathologically and clinically undistinguishable from sPD, *LRRK2* mutation carriers exhibit considerable clinical and pathological variability (Wider et al., 2010). Our results support the hypothesis that mutations in LRRK2 may lead to the formation of the toxic oligomeric forms of α-syn critical for the pathogenesis of PD. However, our findings need to be confirmed in prospectively planned, independent cohort, particularly in samples where PD has been longitudinally assessed such as the ongoing Parkinson’s Progression Markers Initiative.

In conclusion, our current pilot study suggests for the first time that quantification of o-α-syn in CSF has strong potential value as a tool for PD diagnosis and presymptomatic screening of high-risk individuals who are good candidates for clinical trials. However, large-scale, prospective, and well-controlled studies, especially those that include subjects at genetic risk, are necessary to validate the use of CSF o-α-syn as a biomarker for PD.

## Conflict of interest statement

The authors declare that the research was conducted in the absence of any commercial or financial relationships that could be construed as a potential conflict of interest.
